# Neurogenic orthostatic hypotension: the very basics

**DOI:** 10.1007/s10286-017-0437-3

**Published:** 2017-06-15

**Authors:** Horacio Kaufmann, Jose-Alberto Palma

**Affiliations:** Department of Neurology, Dysautonomia Center, New York University School of Medicine, 530 First Avenue, Suite 9Q, New York, NY 10017 USA

## Diagnosis

The diagnosis of orthostatic hypotension (OH) requires blood pressure (BP) readings while supine and upright, either during active standing or during a tilt-table test, to determine the presence of a sustained orthostatic fall of at least 20 mmHg systolic or 10 mmHg diastolic BP. BP and heart rate should be measured after the patient has been supine for several minutes and after standing still (or passively tilted) for 1–3 min. The changes in heart rate on standing help to determine whether the OH is neurogenic in origin. In patients with neurogenic OH (nOH) the increase in heart rate upon standing up is usually <20 bpm. Marked increases in heart rate suggest that the OH is non-neurogenic (Table 1).

**Table 1:** Characteristics of neurogenic and non-neurogenic orthostatic hypotension Modified from [4]

	Non-neurogenic orthostatic hypotension	Neurogenic orthostatic hypotension
Frequency	Frequent (particularly in the elderly)	Rare (<200,000 in the US)
Onset	Variable	Chronic in synucleinopathies. Acute or sub-acute in immune-mediated neuropathies and ganglionopathies
Causes	Intravascular volume loss (e.g., dehydration, anemia) Antihypertensive medications Blood pooling (e.g., large varicose veins, skeletal muscle atrophy) Physical deconditioning, Advanced heart failure Adrenal insufficiency	Defective norepinephrine release from sympathetic post-ganglionic neurons upon standing up
Prognosis	Resolves when underlying cause is corrected	Chronic disorder
Increase in heart rate upon standing	Pronounced (usually >25 bpm)	Mild or absent (usually <20 bpm)
Blood pressure overshoot (phase 4) in Valsalva maneuver	Present	Absent
Increase in plasma norepinephrine levels upon standing	Normal or enhanced (at least ×2)	Reduced or absent (less than ×2)
Other symptoms of autonomic failure	No	Constipation Erectile dysfunction (men) Urinary abnormalities Sweating abnormalities
Concomitant neurological deficits	None (or if present, they are not related to OH)	None Parkinsonism Cerebellar signs Cognitive impairment Sensory neuropathy

## Symptomatic or asymptomatic

**Fig. 1:**
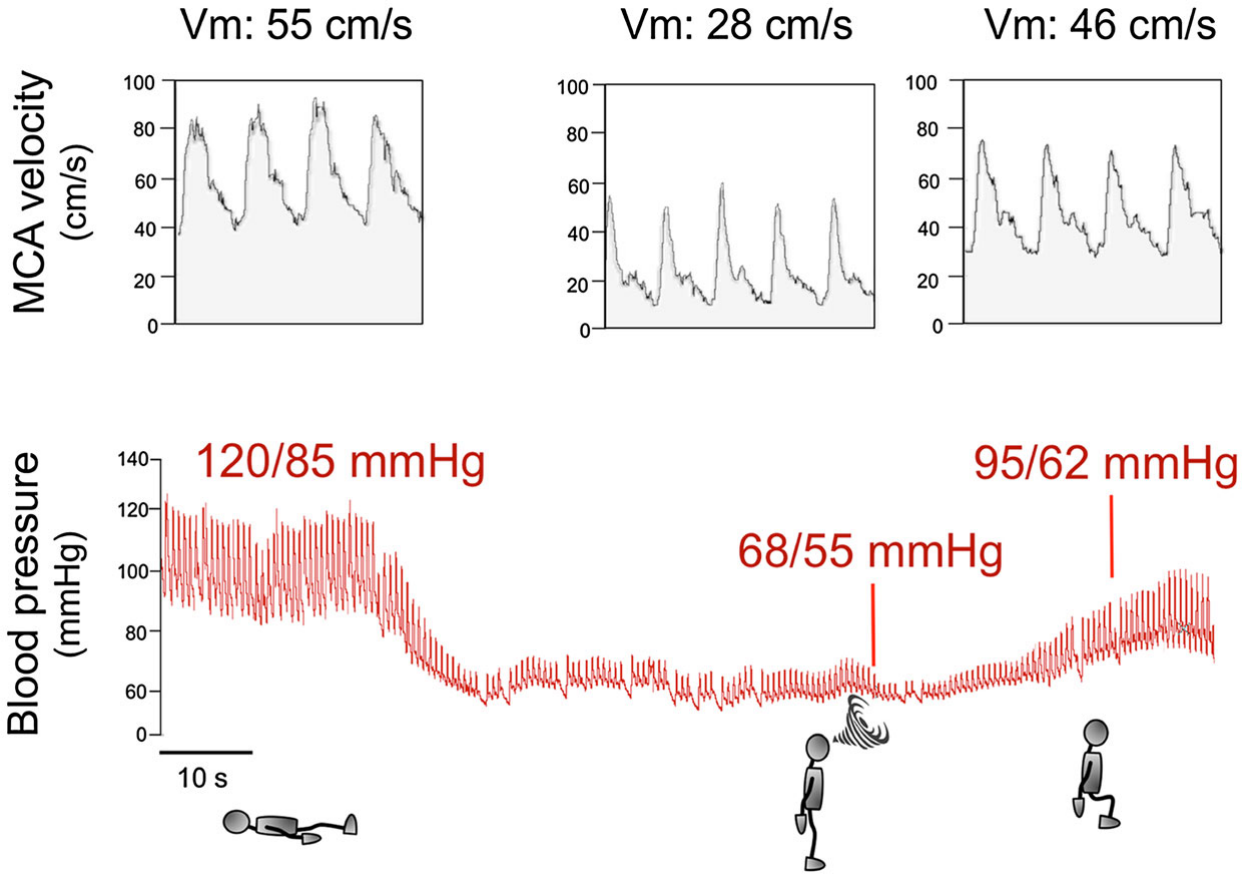
Blood pressure and cerebral blood flow in a patient with neurogenic orthostatic hypotension [4]. The upper tracing displays blood flow velocity as measured by transcranial Doppler ultrasound of the middle cerebral artery (MCA), which is proportional to cerebral blood flow. The lower tracing shows continuous blood pressure recorded with plethysmography. When the patient is in the supine position, BP is normal (120/85 mmHg) and MCA velocity (Vm) is 55 cm/s, indicating normal cerebral blood flow. When the patient stands up, BP drops rapidly to 68/55 mmHg and cerebral blood flow falls by nearly 50% as shown by Vm down to 28 cm/s. The patient becomes symptomatic, feels faint and is unable to remain standing (indicated by a swirl). The patient then sits down and his BP increases to 95/62 mmHg. Although this BP value is still low, the patient is not symptomatic because Vm increased to 46 cm/s, indicating almost normal cerebral blood flow. This tracing shows that for a patient to become asymptomatic, BP does not have to return to normal values but only to increase above the lower limit of cerebral autoregulation

Patients with nOH may or may not have symptoms. Symptoms of nOH typically disappear after the patient resumes the sitting or lying position because cerebral blood flow is restored to levels above the lower limit of autoregulatory capacity (Fig. 1). The chronic nature of nOH allows remarkable adaptive changes in cerebral autoregulatory mechanisms. Indeed, patients with nOH are frequently able to tolerate wide swings in BPs and often remain conscious at pressures that would otherwise induce syncope in healthy subjects [2, 5].

## Ambulatory blood pressure monitoring

**Fig. 2:**
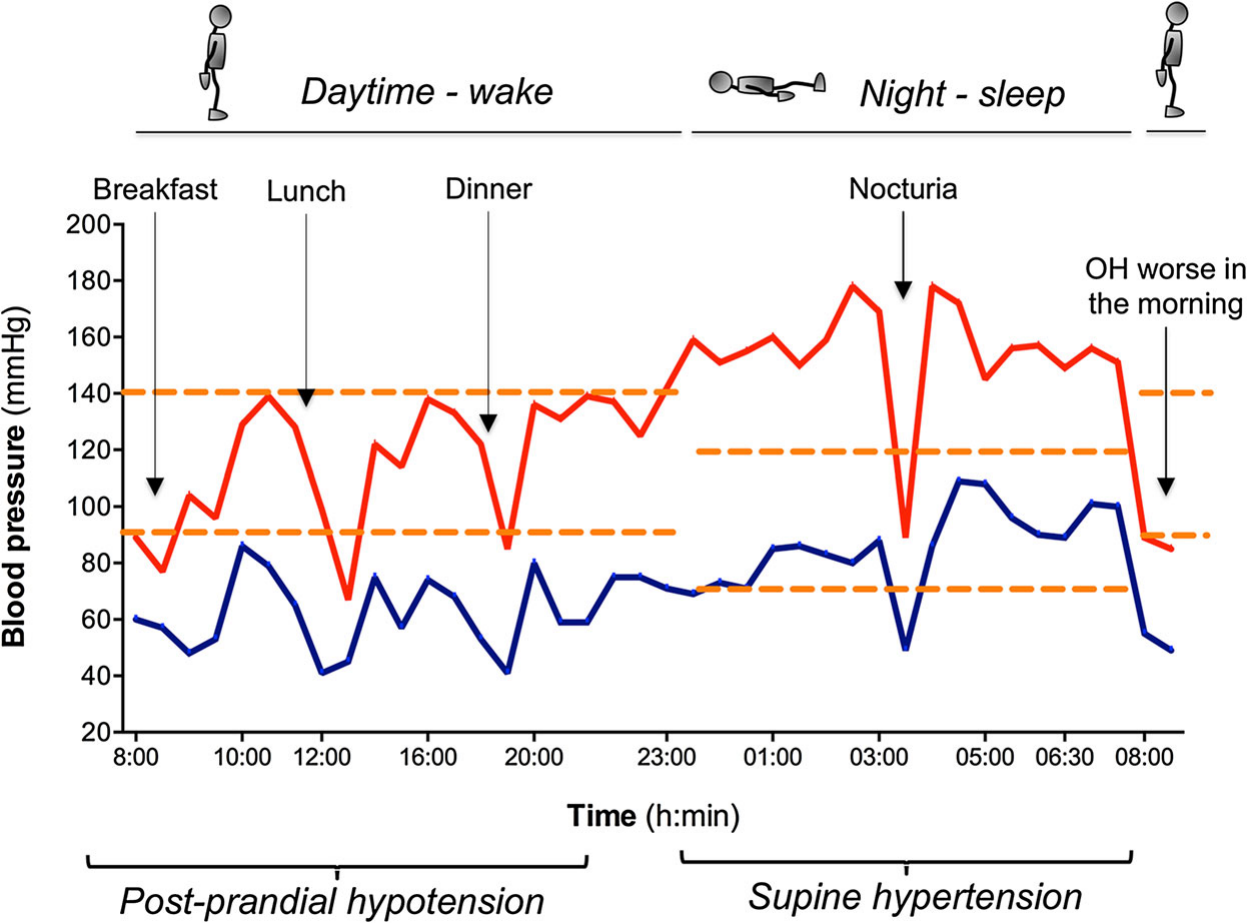
Typical 24-h ambulatory blood pressure (BP) monitoring results in a patient with neurogenic OH and supine hypertension. The *orange dashed horizontal red* denotes the limit for hyper- or hypotension. There is a significant drop in BP (systolic BP <90 mmHg) after breakfast, lunch and dinner (*arrows*). According to this patient’s diary, lunch consisted of a slice of cheese pizza with French fries and a piece of cheesecake, all accompanied by a glass of wine. This is consistent with postprandial hypotension. The patient also had nocturnal hypertension (up to systolic BP of 180 mmHg) while sleeping in the supine position, and one episode of hypotension (while he was in the standing position in the bathroom urinating). Upon awakening the next morning, his orthostatic hypotension is worse due to volume depletion overnight. This recording emphasizes the need to sleep with the head of the bed raised 30°–45° and to avoid high-calorie meals and alcohol during daytime

Ambulatory blood pressure monitoring (ABPM) is useful for the diagnosis of nOH in patients who do not have a fall in BP during an office visit, and to identify post-prandial hypotension and nocturnal hypertension [3] (Fig. 2).

## Management

**Fig. 3:**
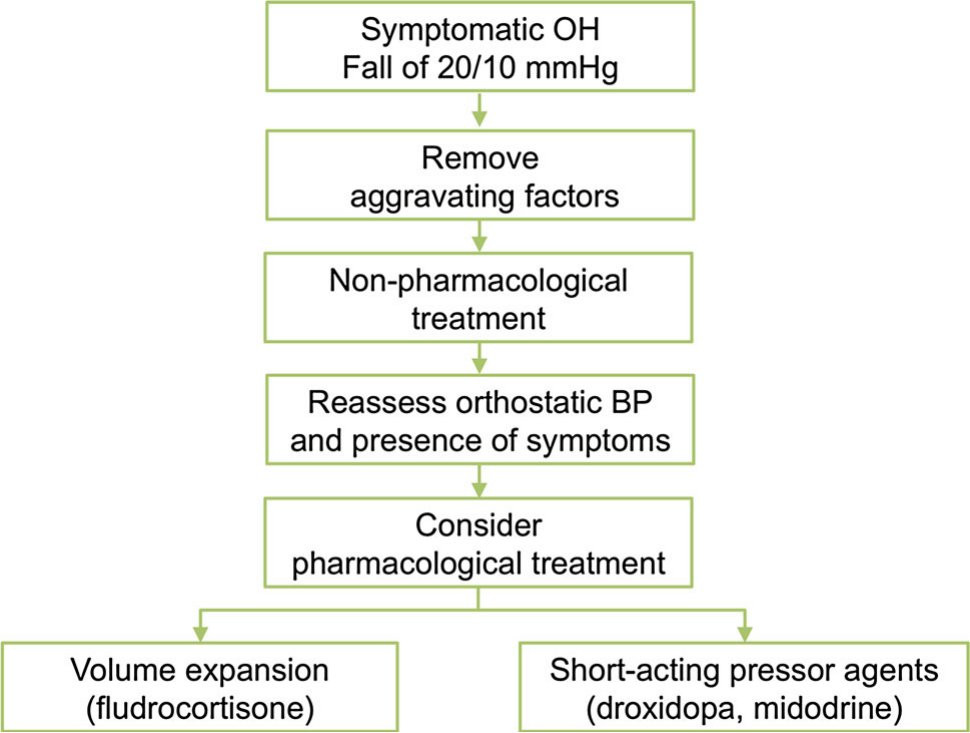
Treatment algorithm in patients with neurogenic orthostatic hypotension. Modified from [4]. Removal of aggravating factors and initiation of non-pharmacological measures must always predate pharmacological agents

The goal of treatment is not to normalize standing BP, but to reduce symptom burden, and to improve quality of life. The steps in management include: (1) correcting aggravating factors, (2) implementing non-pharmacological measures, and (3) drug therapies (Fig. 3).

## Non-pharmacologic management

**Fig. 4:**
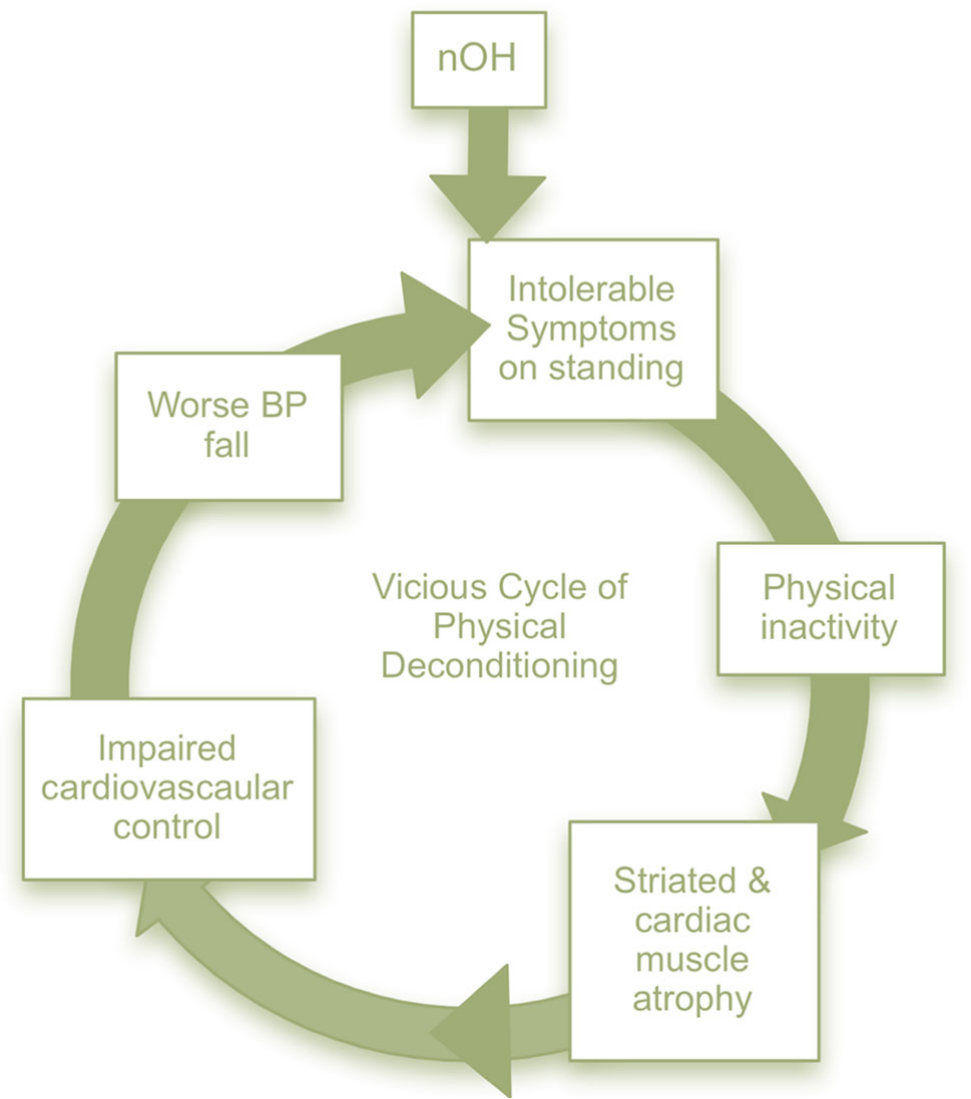
The vicious cycle of physical deconditioning. Modified from [4]. Because patients with neurogenic orthostatic hypotension (nOH) have intolerable symptoms when standing they avoid moving; lack of physical activity results in striated and cardiac muscle atrophy, which further impairs cardiovascular control. This, in turn, further increases the orthostatic fall in blood pressure, worsening symptoms in a perpetual cycle. Improving symptoms can break this cycle

Non-pharmacologic management is vital and often underestimated to ameliorate the symptoms and severity of neurogenic orthostatic hypotenion (nOH). Patients and their families or caregivers should understand the basics of nOH pathophysiology and the importance of non-pharmacologic methods. In many situations, educational materials may be helpful for both the patient and the caregiver (Table 2). Physical inactivity and prolonged bed rest are common in patients with nOH. This leads to cardiovascular deconditioning further worsening the fall in BP and increasing symptoms leading to a vicious cycle (Fig. 4).

**Table 2:** Recommendations of non-pharmacologic treatments for neurogenic orthostatic hypotension Reproduced from [1]

Treatment	Notes
Educate patients and their caregivers	Includes education on understanding orthostatic intolerance; avoiding prolonged standing, immobilization, or prolonged diurnal recumbence; as well as rising gradually from supine and sitting positions, especially in the morning, after meals, and after urination/defecation
Change diet	Eat smaller, rather than large, and more frequent meals. With nOH, sympathetic vasoconstrictor nerve activity is deficient and many patients become severely hypotensive within 2 h of eating. In patients with postprandial hypotension, smaller and more frequent meals are recommended
Avoid increased core body temperature	Elevation in body temperature causes peripheral vasodilation. Patients with nOH should avoid situations that could increase core body temperature, such as excessive high-intensity exercise; exercise when ambient temperature and humidity are high; utilization of hot-tubs, spas, or saunas; prolonged hot showers, etc.
Avoidance of physical deconditioning	Lower body strength training and moderate, non-strenuous activities may be incorporated into standard treatment for patients with nOH
Use head-up position while sleeping	Use of head of the bed elevation up to 30 degrees during sleep. This may reduce nocturia, volume depletion, and supine hypertension
Increase hydration	Increased fluid intake, including rapid water bolus intake if needed, can potentially combat acute nOH symptoms. Volume expansion requires up to 64 oz of water daily. Proper hydration can produce both acute and long-lasting significant clinical benefits to patients with nOH
Increase salt intake	For the patient with nOH, it is recommended that they add up to 1–2 teaspoons (2.3–4.6 g) of salt per day to their normal diet. However, increasing salt intake should be used with caution in patients with heart and kidney failure
Use compression garments	These provide a reduction of peripheral pooling in the lower limbs and splanchnic region. Compression of 30–40 mmHg is required to improve venous return and provide a meaningful blood pressure impact. Abdominal binders offer an effective alternative
Treat anemia and vitamin deficiencies	Anemia leads to decreased blood viscosity and oxygen-carrying capacity and may worsen symptoms of nOH. Vitamin B12 deficiency (<250 pg/mL with elevated methylmalonic acid levels) may also unmask or exacerbate symptoms of nOH

## Pharmacologic treatments

The selection of one drug over the other is related not only to the severity of the patient’s symptoms, but also, in certain situations, based on the clinician’s preference and experience with a certain drug (Table 3).

**Table 3:** Pharmacologic treatments for neurogenic orthostatic hypotension Modified from [1]

Treatment	Recommended dosing regimen	Drug class and notes	Safety notes
FDA-approved for the treatment of symptomatic neurogenic OH			
Droxidopa	100–600 mg three times/day (dosed morning, midday, and 3–4 h prior to bedtime) or tailored to each patients’ needs	Pro-drug of norepinephrine	Supine hypertension, headache, dizziness, nausea, and fatigue; caution in congestive heart failure and chronic renal failure
Midodrine	2.5–15 mg twice or three times/day (dosed morning, midday, and 3–4 h prior to bedtime)	Direct alpha1-adrenoreceptor agonist	Supine hypertension, piloerection, scalp itching, and urinary retention; caution in congestive heart failure and chronic renal failure
Not specifically FDA-approved for neurogenic OH			
Fludrocortisone	0.1–0.2 mg/day; little benefit from observed dose beyond 0.2 mg/day	Synthetic mineralocorticoid. Fludrocortisone is a volume expander that increases sodium reabsorption and enhances sensitivity of alpha-adrenoreceptors	Supine hypertension, hypokalemia, renal failure, and edema; caution in congestive heart failure
Pyridostigmine	30–60 mg twice or three times/day	Acetylcholinesterase inhibitor. Marginal efficacy in nOH	Abdominal cramps, diarrhea, sialorrhea, excessive sweating, urinary incontinence

## Treatment of supine hypertension associated with neurogenic orthostatic hypotension

Hypertension in the supine occurs in ~50% of patients with nOH. There are no controlled clinical trials on its treatments. In patients with nOH experiencing supine hypertension [systolic blood pressure (BP) of 160–180 mmHg or diastolic BP of 90–100 mmHg], there is agreement that sleeping with the head of the bed raised at least 30°–45° should be recommended. If patients are experiencing sustained severe supine hypertension (systolic BP of >180 mmHg or diastolic BP of >110 mmHg) even after sleeping in the semi-sitting position, some experts advocate using short-acting an antihypertensive agent before bedtime (Table 4). This remains controversial and clinicians must be aware that pharmacological treatment for supine hypertension increases the risk of worsening hypotension and falls when the patient gets up at night or in the early morning.

**Table 4:** Pharmacological treatments for supine hypertension associated with neurogenic orthostatic hypotension Modified from [1]

Treatment options^a^	Typical dose
Captopril	25 mg at bedtime
Clonidine^b^	0.1 mg with dinner
Hydralazine	10–25 mg at bedtime
Losartan	50 mg at bedtime
Nitroglycerine patch	0.1 mg/h patch at bedtime (remove patch in the morning)

No controlled trials have been performed. The risk–benefit ratio should be individually assessed

^a^Short-acting antihypertensives should be administered at bedtime only, not during daytime hours. Many medications have twice or three times/day as recommended dosing and patients may inadvertently start taking these medications during daytime hours and worsen symptoms of nOH

^b^The use of clonidine carries a risk of a severe hypotension in the morning as well as rebound hypertension
